# Video-Algorithmic Patient Monitoring in Mental Health Inpatient Settings: Qualitative Study of Patient or Consumer, Clinician, and Vendor Perspectives

**DOI:** 10.2196/85518

**Published:** 2026-07-20

**Authors:** Piers Gooding, Hamilton Kennedy, Simon D'Alfonso, Timothy Kariotis, Catherine Daniel, Bridget Hamilton

**Affiliations:** 1 La Trobe Law School, La Trobe University, Bundoora, Melbourne, 3086, Australia, 61 3 9479 1846; 2 Centre for Mental Health Nursing, The University of Melbourne, Melbourne, VIC, Australia; 3 School of Computing & Information Systems, The University of Melbourne, Melbourne, Australia; 4 The University of Melbourne, Melbourne, Australia; 5 School of Nursing, Midwifery and Paramedicine, Australian Catholic University, Melbourne, Victoria, Australia

**Keywords:** vision-based patient monitoring and management, passive remote monitoring technology, video-algorithmic patient monitoring, monitoring and surveillance in mental health settings, health and artificial intelligence

## Abstract

**Background:**

Video-algorithmic patient monitoring (VAPM) combines remote, noncontact sensors and algorithmic analysis and is increasingly trialed in acute psychiatric and other care settings. While promoted for improving safety and reducing risk, it raises ethical concerns regarding safety, privacy and surveillance. Little is known about how those encountering VAPM in mental health care contexts anticipate its use and potential impacts, including where it has not yet been implemented.

**Objective:**

This study aimed to explore the views of patients or mental health consumers, specialized mental health nurses and nurse academics, hospital managers, and technology vendors regarding the appropriateness and anticipated implications of VAPM in mental health inpatient care.

**Methods:**

This qualitative study identified key stakeholders in Australia via networking techniques for participation in a deliberative workshop. A deliberative workshop was held, and the workshop discussion was audio-recorded, transcribed, and thematically analyzed, consistent with methods in health technology research, which enable exploration of different viewpoints, including convergences and divergences across stakeholder groups.

**Results:**

In total, 16 stakeholders participated, exploring themes concerning (1) contestation over the rationale for VAPM in mental health settings, (2) VAPM reshaping care and relationships, (3) perceived harms of VAPM, (4) perceived observational support for safety and reduced disruption, (5) serious privacy implications of VAPM, (6) the need for appropriate governance, and (7) the potential for VAPM to transform, not augment, service delivery. General views differed across groups. Patients or service users expressed concerns about privacy, coercion, and the potential to intensify stigma. Mental health nurses were cautious but interested in possible benefits for safety and suicide prevention. Hospital managers and technology vendors largely emphasized safety gains.

**Conclusions:**

The findings suggest that the anticipated risks of VAPM are primarily experienced subjectively, as infringements on privacy, dignity, and trust, while purported benefits remain largely untested and unquantified. From a utilitarian perspective, direct comparison is therefore difficult—the risks are set out in the anticipated experiences of those with lived experience, and the benefits remain hypothetical. From this view, robust, independent evidence of real-world outcomes is required. Yet, for some participants, the very premise of such calculation was rejected, with privacy, dignity, and trust regarded as nonnegotiable, rather than items for trade-off. If VAPM is to be pursued at all, it should proceed only with extreme caution, with transparent evidence of outcomes, and with meaningful participation from those whose lives and care are most directly impacted.

## Introduction

Technology used to monitor and surveil is proliferating in many areas of contemporary life. However, there are not always opportunities for people to consider the implications, personally and socially, for monitoring and surveillance in specific settings.

This paper refers to “video-algorithmic patient monitoring” (VAPM) to describe a form of monitoring that incorporates remote sensors, video technology, and algorithmic analysis. This technology is being trialed in varied health and social care settings, including mental health inpatient wards. VAPM involves video cameras or other sensors, artificial intelligence (AI) software, and an interface for nurses and other mental health practitioners to make remote, noncontact clinical observations and data collection about patients in hospital environments [1]. Other descriptors include “vision-based patient monitoring and management” and “passive remote monitoring technology” [2,3].

We conceptualize this monitoring arrangement as a “sociotechnical system” insofar as it is a “system involving the interaction of hard systems and human beings, in ways that either cannot be separated or are thought to be inappropriate to separate” [4,5]. This framing offers a way to analyze the dynamic interaction of people and technology, rather than narrowly focusing on the technology itself.

Traditional patient monitoring in hospitals involves in-person checks by health care professionals or simple alerts provided by clinical technology that tracks certain vital signs (eg, a pulse oximeter). VAPM is an iteration of these monitoring techniques. Proponents advocate for their use to improve upon in-person monitoring, including traditional forms of vital sign monitoring, and to reduce disruption for patients. Others have raised concerns that these technologies pose a threat to service user safety, privacy and dignity [6,7].

In mental health inpatient settings, VAPM involves patients’ bedrooms being fitted with sensors [1], typically designed to focus on a person’s pulse, breathing rate, and movement. Signals are detected remotely and continuously, based on physiological phenomena such as the chest rising and falling or the use of photoplethysmography (a simple optical technique that uses light to measure blood volume changes in peripheral circulation). Unlike vital sign monitoring, which is used extensively in health care settings, VAPM entails remote monitoring that does not rely on sensors attached to a person’s body (on-body sensors). Attempts are underway to use such data as alerts to reduce self-harm, assault, and suicide in mental health wards [8].

At the time of data collection (December 2021), there was limited research on the impacts and use of VAPM [1,2]. Although several studies emerged since this time [9-13], empirical studies into VAPM remain limited. A systematic review of surveillance-based technology initiatives in inpatient and acute mental health settings [14] identified 9 studies that covered VAPM (the systematic review authors used the term “Vision-Based Patient Monitoring and Management” [14]), all from the United Kingdom; all were rated as “low quality,” except one, which was rated “high quality” [3]. In total, 8 of the 9 studies also reported conflicts of interest, with the high-quality study being the only paper by Dewa et al [3], which did not report any conflicts of interest [14]. As we will discuss, the post-2021 studies—which are UK-centered—are broadly consistent with participants’ anticipation that VAPM might reduce disturbance from in-person observations and improve efficiency (particularly overnight), with potential flow-on safety and cost implications [8-11,14]. However, participants also anticipated that these benefits would remain contestable and difficult to justify without clear evidence and strong governance, and that concern is reinforced by the still-limited and often conflicted UK evidence base [14].

The stated aims of VAPM include reducing disturbance to patients by making observations less intrusive, which may have several flow-on benefits to people’s health and recovery [1], improving efficiency with night-time safety and reducing reliance on intensive monitoring, which may lead to reduced costs [10], improved quality and safety in care [10,11], reduction in incidents of assaults, and reductions in the associated involuntary tranquilization of patients and service users [13].

Ethical and legal concerns have arisen in the United Kingdom, with several complaints about the technology being reportedly made by rights organizations, nurses, and patient groups on the basis that VAPM raises privacy concerns, is objectionable, and is potentially harmful to patients [6,15]. In at least 1 National Health Service (NHS) Trust, complaints reportedly led to a cessation of VAPM [16]. These concerns suggest that even if further studies confirmed benefits to patients’ sleep or other areas, such as reduced self-harm or suicide, such findings would have to be considered alongside other social, ethical, and legal implications of the technology. The legal implications of VAPM, for example, are not well understood [17].

This paper reports the findings of a qualitative exploratory study, via a workshop with key stakeholders which asks about the anticipated implications of VAPM in mental health inpatient settings. The study occurred in Australia, where VAPM is not widespread but has been applied in aged care settings [18] and seems likely to be trialed in mental health care settings [19]. The study aims to consider not just the practical and clinical implications of VAPM, but also the social, ethical, and legal issues, situating VAPM within broader debates about surveillance technologies in health care and their effects on privacy, consent, trust, and so on. This paper also draws on recent scholarship on new technologies of automation, such as AI ethics and algorithmic accountability, to analyze how VAPM may impact governance, decision-making, and trust in inpatient mental health care. We use these perspectives to assess the implications of data-driven monitoring for matters such as privacy, agency, and relationships of care.

## Methods

### Overview

We adopted a deliberative workshop format to elicit multistakeholder perspectives on the use of VAPM in mental health inpatient settings. Methods such as citizens’ juries, co-design workshops, and deliberative panels are increasingly used in health technology and medicolegal research to qualitatively explore ethical, legal, and social implications of new technologies [20,21]. They enable structured engagement with complex dynamics and trade-offs concerning issues of privacy and safety and are supported by recent empirical examples demonstrating their value in AI health care deliberation and digital tool development [22,23].

An online workshop was held in December 2021 with 16 participants and the 6 study authors, as well as an additional research assistant and an independent facilitator. The authors worked with an independent facilitator with expertise in group process design to develop an online workshop that was interactive and would provide participants with the opportunity to engage with evidence, deliberate, and respond both in verbal and written formats.

### Recruitment

Using networking techniques, each coauthor identified 4 individuals who were invited to the workshop in a process of purposive sampling, informed by criteria concerning participants’ appropriateness, capacity, and willingness to participate [24]. We aimed to recruit participants from 5 broad groups: consumers, nurses and nurse academics, health care managers, technology vendors, and ethico-legal and psychology academics. Invitations were sent directly to participants via email. As academics, practitioners, and advocates who research extensively in the field, we drew on our networks, as well as “cold-calling” potential participants to meet recruitment targets.

### Participants

The 16 participants included 3 mental health consumers, 7 specialized mental health nurses (employed across direct care and academic and policy roles), 2 managers of acute mental health care services, 2 patient monitoring technology vendors, 1 legal academic, and 1 public health academic who specializes in inpatient hospital settings. The research team included a consumer academic, a legal academic, a computing academic who specializes in digital mental health, 2 mental health nurse academic researchers, and a health informatician.

Given that patients are most directly impacted by VAPM, the authors prioritized the recruitment of mental health “consumers,” a term used in Australia to refer to someone who has used or continues to use mental health care services. (“Patient” is sometimes used throughout this paper, acknowledging that involuntary patients in mental health inpatient settings do not choose to be there in the sense conveyed by “consumer.”) We sought a diversity of consumer perspectives on the topic of VAPM [25], but only 3 were able to attend the workshop, all of whom had served in representative and advocacy roles that required them to understand and articulate the views of diverse consumers. This latter experience enabled them to engage with both the governance and policy dimensions of VAPM and to consider VAPM based on their personal experience of inpatient mental health settings. A total of 7 consumers were invited to participate, all of whom expressed interest, but only 3 of whom were able to make the scheduled workshop.

We also prioritized the recruitment of nurses, given the implications of VAPM for care work. Psychiatrists and health service managers were invited, as they would be expected to make resourcing and care design decisions and interact with VAPM in mental health inpatient settings. Two vendors of VAPM who worked in a private technology company were invited, given technology vendors are involved in the creation, sale, and implementation of these technology types. Several scholars of health law and ethics were invited, but did not reply or could not attend, except for 1 legal academic.

The participants have been coded according to their stakeholder group, which is presented in Table 1.

**Table 1.** Participant codes and stakeholder groups.

Participant or researcher code	Participant group
P1	Technology vendor
P2	Consumer
P3	Health service manager
P4	Nurse
P5	Health informatics academic
P6	Legal academic
P7	Consumer academic
P8	Nurse
P9	Nurse academic
P10	Technology vendor
P11	Consumer
P12	Nurse academic
P13	Nurse
P14	Nurse academic
P15	Health service manager (with a clinical background)
P16	Nurse academic

### Ethical Considerations

This study received ethics approval from the University of Melbourne Ethics Committee (reference: 2021-22674-23512-4). All participants provided written and verbal informed consent before participating in the workshop. Participants were informed about the purpose of the study, the nature of their participation, and the intended use of their contributions, including publication of anonymized findings. Participation was voluntary, and participants could decline to answer questions or withdraw from the study prior to publication. To protect participant privacy and confidentiality, all contributions were anonymized, and participants are identified only by participant codes and broad stakeholder groups in the paper. No identifying information is reported. Participants were offered an Aus $50 (US $34.50) honorarium to participate if they were not able to otherwise attend in a professional capacity.

### Reflexivity and Researcher Positionality

All authors approached VAPM without settled views and with an exploratory orientation. One author (the legal academic) had previously written about the ethico-legal issues raised by VAPM [17], and the consumer academic’s interest in the topic started with experience serving on a health service advisory group that had been asked to share views on VAPM. Reflexivity was addressed through ongoing team discussion, with attention throughout to separating normative concerns about VAPM from faithfully reporting participant accounts. Throughout the analysis, we aimed to let the data guide theme development, while acknowledging that researchers’ standpoints shape interpretation and therefore warrant explicit disclosure rather than being glossed over.

### Data Collection

The 3-hour workshop was moderated by an independent facilitator, with breakout discussions in small groups facilitated by the 6 authors. The workshop first entailed a plenary session in which members of the research team summarized existing empirical research on the use of the monitoring systems in acute psychiatric settings. Participants were provided information before the workshop, which included a summary of the literature on VAPM. Participants were separated into small groups and asked to share their views. Each group comprised a diverse range of participants (rather than being separated into cohort groups).

The small group discussions were an adapted form of focus group, a method offering rich descriptions by relevant actors of a phenomenon and the context in which it operates [26]. Breakout discussions were facilitated with the aim of offering a neutral, semistructured approach. Facilitators sought to guide the discussion to remain focused on the workshop prompts while refraining from evaluative commentary on the merits of particular viewpoints.

The audio recording of all workshop group discussions was transcribed for analysis. In total, 5.5 hours of audio-recorded data were generated.

### Analysis

Analysis proceeded using thematic content analysis to capture broader group-level themes across the data [27], and a descriptive phenomenological method to frame and analyze the subjective, experiential accounts from workshop participants (particularly if they shared personal narratives, which occurred with some consumers and mental health nurses). Descriptive phenomenology takes an interpretivist stance, well-suited to practice-oriented research that seeks to record and understand participants’ lived experience of a phenomenon while suspending researcher assumptions [28].

Four of the coauthors (SD, BH, TK, PG) used a blended approach of inductive and abductive coding [29] to code the workshop transcripts. This was done in person (not using coding software), using printed transcripts, and codes were written on sticky notes before being grouped into themes on a physical whiteboard. Each transcript was read by at least 2 researchers to ensure coding consistency. The research team met to compare interpretations, discuss points of divergence, and refine the developing thematic structure.

Differences in coding were resolved through discussion and consensus. Consistent with a reflexive thematic analysis approach, we did not calculate a formal statistical measure of intercoder reliability. Rather than seeking statistical reproducibility, our aim was analytic rigor through collaborative scrutiny, reflexive dialogue, and iterative theme development.

Quotes were selected to exemplify themes. Participants did not review findings before publication.

We did not aim to achieve thematic saturation in the sense of exhaustively capturing all possible views on VAPM. Rather, the purpose of the workshop was exploratory, designed to elicit and compare the reasoning, concerns, and anticipated benefits articulated by key stakeholder groups and to identify points of convergence and contestation relevant to decisions about VAPM, including whether it ought to be used, and if so, how it should be governed. In qualitative approaches aimed at interpretive thematic analysis and exploratory issue-mapping, “saturation” can be a poor fit where the aim is to illuminate key tensions and meaning-making rather than to enumerate all views [30].

## Results

The analysis led to the development of 7 themes, some of which included subthemes. In Table S1 in Multimedia Appendix 1, we provide illustrative quotes for the cross-cutting theme and each major theme and subtheme to convey the findings overall, before summarizing each.

### Contestation Over the Rationale for VAPM in Mental Health Settings

A recurring thread across the findings was contestation over the rationale for using VAPM in acute psychiatric settings and its unique implications in the mental health care context. A fundamental tension appeared in the discussion between the utilitarian ambition for enhanced safety and efficiency (often driven by administrators and technology vendors) and what might be described as a relational ethics of care approach, which prioritizes the ethical imperative to protect human dignity, privacy, and the relational essence of care (strongly advocated by consumers and many nurse and academic participants).

A utilitarian account of the technology considers its potential as a versatile tool that provides an “additional layer” of monitoring and “visibility” (Participant 10) in scenarios where continuous human observation is not feasible. From this view, the technology has the capacity, principally, to enhance patient safety, reduce physical disruptions, and provide valuable operational support to clinical staff. Those sympathetic to this view highlighted its potential utility in proactively identifying disturbed sleep patterns, detecting medical emergencies, and enhancing safety measures against potential harm from other consumers or staff—and thus viewed it as potentially amenable to ethical and high-quality care. From this view, VAPM can support and extend the reach of clinical care, particularly when direct physical presence is limited.

However, most participants focused on the potential negative effects of VAPM. Some—particularly the consumers—expressed profound concerns that it represents a dehumanizing, intrusive, and potentially harmful form of surveillance that undermines the core principles of high-quality, contemporary mental health care. Key aspects of this account include invasion of privacy, exacerbation of distress, erosion of trust and therapeutic relationship, potential for misuse and lack of agency, and complacency and deskilling of staff. Consumer participants with this view expressed a profound rejection of the technology in patient bedrooms.

These 2 broad accounts seemed to be underpinned by differing accounts of the ways in which the mental health care context was seen as unique compared to other areas of medicine. Most participants, particularly consumers and nurses, queried whether a tool primarily designed for physiological monitoring would align with the distinct needs and expectations of mental health care, where therapeutic engagement and trust are paramount. For example, one consumer said:

There’s no electronic monitoring that will tell you that my hope was just crushed [...] The only way you can know that we’re thinking and feeling that is to talk with us and listen to us.

[Participant 2]

A nurse (Participant 4) felt the proposal aligns with an “overly restrictive non-therapeutic program” and moves away from “strengths-based consumer-led models of care.”

In contrast, a hospital manager (Participant 15) raised a comparison with general intensive care units, noting that in physical hospital care settings, staff “don’t blink an eye” at monitoring physiological data. He added, “[a]s a rule, we’ve never had that facility in our ICU settings” within mental health, implicitly suggesting that the disparity in technological monitoring between the 2 creates a disparity in the safety and quality of care. Here, mental health care was viewed as different because it did not have parity of care with general health, which VAPM could help remedy.

All participants acknowledged the potential for coercion in public psychiatric hospital units, with several participants noting that this technology will have unique implications, given that patients or consumers are already significantly disempowered and monitored in (often locked) mental health care contexts.

### VAPM Will Reshape Care and Relationships

#### Overview

An overarching theme among participants was the impact of VAPM on care and the people who give and receive it. Most participants expressed a concern with the potentially dehumanizing impact of monitoring and surveilling people’s bodies for “signs” and movements, instead of asking people about their distress or checking on them face-to-face. The extent to which VAPM was indeed viewed in this way varied in three ways, between (1) those who felt certain it was dehumanizing, (2) those who felt it potentially dehumanized but that more detail was required alongside consideration of potential benefits, and (3) those who felt it was a potentially exciting opportunity to transform care, and indeed to “rehumanize” care by freeing up care professionals’ time for more valuable face-to-face care.

#### VAPM May Dehumanize Care Depending on How It Is Used

Participants raised competing concerns about whether VAPM would support or undermine care.

In total, 3 participants expressly described VAPM as “dehumanising” (Participant 2, Participant 4, and Participant 13). One participant said it felt “grotesque” and “the complete opposite direction that we should be heading for good care” (Participant 2). Such accounts conveyed not only fear of misuse but also anger at the idea that replacing observation with surveillance contradicts the relational ethos of mental health practice. Participants also worried that remote viewing could reduce therapeutic contact and intensify privacy intrusion; during in-person monitoring, a patient could at least know when they are being observed, unlike remote continuous monitoring.

Concerns extended beyond consumers to staff. One nurse warned that VAPM may be “dehumanising for the person you’re serving, but also the workforce” (Participant 4), moving practice away from strengths-based and consumer-led approaches. For these participants, the possibility that biometric monitoring could detect crises did not outweigh the loss of direct contact, trust, and the complex facets of human care.

#### VAPM Could Humanize Care by Creating More Time for It

Some participants suggested that VAPM could actually create conditions for more humanized care. By automating routine and time-consuming observation tasks, the technology was seen as potentially freeing staff to focus on therapeutic interaction. A nurse academic explained that much of the current workload “is really not about actually caring for the person,” but if monitoring was managed by technology, staff could “use our time and resources to actually support the person in a more therapeutic way” (Participant 14). This was echoed in specific clinical contexts, such as eating disorder units, where constant physical checks can be disruptive; VAPM was imagined as a way to reduce unnecessary physical intrusion while still safeguarding health.

Others envisioned VAPM as a “backup” safety mechanism that might enable staff to spend more time present on the ward. A hospital manager (Participant 3) linked this possibility to wider efforts to design units that keep staff “out there with the consumers” rather than tied to nursing stations. In this framing, the technology could reduce the burden of repetitive checks while fostering closer interaction, more group activity, and ultimately safer environments by reducing suicides (discussed in the VAPM as Observational Support for Safety and Reduced Disruption section). These perspectives framed VAPM not as a substitute for relational care, but as a tool that might support and expand it, if used judiciously.

#### Consent to Being Monitored

Some participants proposed that VAPM could be made more acceptable—and thus more “humanizing”—through a clear consent process, giving patients the choice to opt in or out depending on their needs. A nursing academic (Participant 12) suggested that for some, the technology might be preferable to the disruption of in-person checks during the night, allowing people to choose “what’s going to be better for me.” In this framing, VAPM could be a tool of choice, reducing intrusive physical monitoring and supporting therapeutic rest where it aligned with a consumer’s comfort and sense of safety.

Yet, these proposals for consent were met with significant skepticism and distrust by some. Consumers worried that promises of choice would not be honored in practice: one participant doubted whether the system would truly be switched off if they declined and feared it could be misused by staff, undermining any sense of agency (Participant 2). Others warned that systemic pressures, such as staff shortages, could lead to VAPM becoming a default, regardless of individual wishes (Participant 11 and Participant 4).

Contestation also arose over whether consent requirements should differ from current observation practices, with a vendor questioning why AI monitoring should need explicit permission when in-person night checks do not (Participant 10). Nurses responded that, unlike a glance through the door, VAPM automatically records physiological data, which in other contexts would require active consent for a patient with capacity (Participant 12). For one nurse academic, the idea that VAPM should be installed so there was “no way of avoiding it,” and that this could be presented as a “selling point” was described as “very disturbing” (Participant 9). Taken together, these perspectives highlight potential issues with relying on consent as a bridge to acceptability in inpatient settings, where structural constraints and fears of surveillance may undermine aspirations for genuine choice. The distinct privacy implications of VAPM (including ‘privacy of place’ and ‘privacy of data’) are addressed in the section “VAPM Has Serious Privacy Implications.”

### Perceived Harms of VAPM

There was broad agreement that VAPM could cause harm, though participants differed on the extent of this harm and how it compared to potential benefits. Consumer participants, along with several nurses, voiced the strongest reservations. For some, the concern was visceral: Participant 5, a public health academic who specializes in researching inpatient settings, described her “gut” as:

really uncomfortable ... if I was in that position, I wouldn’t like it. I wouldn’t like the experience of having a video camera in a place where I was sleeping.

Nurses also expressed apprehension, with Participant 4 warning of “human rights and ethical considerations and safety,” a theme echoed by others.

Several participants argued that VAPM could in fact undermine, rather than enhance, safety. One consumer explained:

I’d be trying to hit it with the chair, put water on it to anything I could to damage it because I’d be I’d feel really unsafe from it. The more distressed I was, the more I’d feel like that.

[Participant 2]

She added that surveillance might not stop self-harm, but simply shift it elsewhere: “if there’s monitoring in the bedroom, then I’d self-injure on the toilet, you know.” A nurse (Participant 13) cautioned that the technology could encourage “complacency” among staff, reducing engagement on the assumption that safety was assured. One of the vendors (Participant 1) acknowledged the same risk, noting that staff might fall into a “false sense of security” and miss critical issues.

Participants also highlighted the possibility of secondary and longer-term harms. Participant 7, a consumer, warned, “If I went into a ward that had these in every single bedroom, I’d probably flip lid.” Both consumers and nurses suggested that VAPM could “exacerbate paranoia” (Participant 7), “play into ... someone’s mental state” (Participant 1, technology vendor), or worsen anxiety. The technology was also described by two participants as potentially retraumatizing (Participant 12 and Participant 13), particularly for people with experiences of domestic violence in which surveillance had been used as a tool of abuse. Others raised concerns that awareness of such monitoring might deter people from seeking help in services where VAPM is in place. Some participants thus framed privacy intrusion as a harm in itself and a source of distress and mistrust. Detailed privacy implications (privacy of place and privacy of data) are addressed in theme “VAPM has serious privacy implications.”

### VAPM as Observational Support for Safety and Reduced Disruption

Participants who saw potential benefits framed VAPM as a way to support observation in two related ways: (1) risk detection or prevention and (2) less disruptive routine checks, especially overnight. Comments included that the monitoring offers an “exciting opportunity if it’s used well” (Participant 14), provides an “opportunity to augment our care” and “prevent deaths” (Participant 15), and helps provide “continuous monitoring ... [and] constant data feeding back” (Participant 13).

#### Preventing Rare but Severe Adverse Events

Some participants argued that VAPM could add an extra layer of visibility to help detect and prevent rare but serious events, including suicide, sexual assault, or medical deterioration. Participant 15 (hospital manager) stated that although rare, deaths in hospitals “do occur frequently enough” and said his “primary concern is always the safety of the consumers or patients”:

some of our patients unfortunately pass away in the middle of the night ... And of course, the other issue is the suicide risk, in the night. So the extent that this technology may help us manage prevent deaths, I think that’s an important consideration*.*

Participant 13 (nurse) highlighted the importance of mitigating interpersonal risks, particularly for vulnerable consumers, stressing the need for “sexual safety” and “being able to ensure that there’s not a person entering another person’s bedroom. I feel like that would offer significant reassurance to particularly our female consumers.”

Physical health monitoring was seen as potentially beneficial when predicting, preventing, or responding to someone having a physical medical episode (eg, a stroke, fall, or asthma attack) or attempting suicide. Participant 15 (hospital manager) explicitly mentioned preventing deaths from issues like respiratory depression and managing suicide risk at night as key concerns occupying his mind, stating that “[a]s a clinician, this is what makes me deeply uncomfortable and I think this is an opportunity to augment our care.”

#### Reducing Disruption From Routine Observation

Others focused on using VAPM to reduce sleep disruption and intrusive checks while still supporting physical monitoring. Participant 16 (nurse academic) noted that consumers frequently comment about the “very untherapeutic environment that nighttime [routine observations of every consumer] have on people and that disruption to sleep” and invasion of privacy, suggesting that the technology could offer “some positive aspects to that.” Participant 13 (nurse) said that “lack of overnight disruption would be great, too.” Participant 12 (nurse) agreed and described current night checks as “terrible,” recalling that staff “always feel really bad” about waking patients up and shining a torch on them.

### VAPM Has Serious Privacy Implications

Most participants raised concerns about intrusions on privacy. Participants distinguished between privacy of place—bedrooms and other intimate spaces—and privacy of data—the capture, storage, access, and potential secondary uses of video and physiological information.

#### Privacy of Place: VAPM Reshaping Privacy in Intimate Spaces

It is accepted that patients within inpatient mental health settings are subject to significant observation, even to the extent that observation is the primary objective of some hospital admissions. However, participants viewed VAPM as enabling observation of a different nature and scale, with heightened risks of intrusion and misinterpretation.

A participant warned that VAPM could intrude on “a personal moment” (Participant 8, nurse) and “a private intimate moment” (Participant 9, consumer), such as masturbation or other personal activities. Without control over when monitoring occurs, observation becomes constant and intrusive, and misinterpretation of what is seen could negatively affect the care someone receives.

Participant 15 (hospital manager) suggested that privacy and confidentiality risks could be mitigated by limiting the visual detail available to staff, for example, by displaying only a “pixelated body form” (sometimes referred to as a “point cloud”) rather than a live video feed or detailed image of a person “sleeping or going to the toilet or whatever and doing something private.”

#### Privacy of Data: Handling, Retention, and Secondary Use

Concerns about data handling, storage, accessibility, and the eventual use of the generated consumer data were widely discussed among participants, demonstrating apprehension closely related to privacy and surveillance. Consumers expressed fears about how information could be retained and later misused, with one saying “[W]e hear lots of examples of from consumers of incidences from 10, 15 years ago being brought up now ... that affects their treatment now” (Participant 8) and querying whether that might occur with data collected by VAPM. Others questioned whether routine physiological data collected through VAPM would become part of a person’s permanent medical record, with implications for how future admissions or treatments might be judged.

Nurses and consumers raised uncertainties about where and how the data would be used and stored, including whether it could be subpoenaed or whether it might be used in staff performance management, likening the situation to “police walking around with our cameras on” (Participant 9). Beyond immediate clinical settings, participants worried about the commodification of data, even in deidentified form. As one consumer academic put it, such datasets could be sold or repurposed to create behavioral profiles, with the risk of reinforcing surveillance and targeting of certain groups (Participant 11). Together, whether these perceptions were correct or not, these sentiments reflect concerns about how data generated by VAPM may pose risks not only for individual privacy but also for equity, trust, and the long-term integrity of mental health care.

### VAPM Requires Appropriate Governance

#### Procedures and Guidelines to Support Use of VAPM

Most participants emphasized the importance of carefully developed guidance and rules on use, should a VAPM trial proceed. A nursing academic described the need for “strict guidelines necessary on how it supplements care rather than replacing care,” which “would need to be really clear [as to] the expectations on staff on how it was to be used appropriately” (Participant 16). Concerns were also raised about responsibility in the event of a critical incident. A consumer (Participant 11), who had a legal background, noted that good “regulation and governance” must be in place, as these new technologies “stretch the existing structures that we have” and may even create liability under legal obligations that require a certain standard of care (evaluating the accuracy of this claim is outside the scope of this paper). A hospital manager described the need for a “fairly robust procedural implementation process to make sure that people’s concerns were addressed” (Participant 15).

Legal and clinical participants worried that because the technology is proprietary, its inner workings would remain opaque, preventing proper scrutiny or challenge. Participant 6 (lawyer) noted that descriptions of the technology seem to be “always really vague.” She questioned the nature of the AI and algorithm behind VAPM, stating that the inner workings of privately developed technologies more generally, which are sold to health services, are:

not explained because it’s proprietary. Someone owns it and so on, which then makes it even harder to kind of scrutinise, let alone to kind of challenge if something goes wrong.

Liability was thus not only understood in terms of potential harm in individual cases, but also as matters of organizational governance and responsibility: managing sensitive data appropriately, ensuring adherence to staff protocols, establishing private sector accountability mechanisms, and safeguarding the human rights of vulnerable consumers. Participants also questioned who would authorize the technology, how it would fit within established models of care, and whether it would support or displace existing clinical practice. No clear answers emerged, but several participants emphasized that such technologies should ultimately support, rather than replace, the work of health care professionals.

#### Safeguards in the Adoption of VAPM

Participants who supported trialing VAPM generally agreed on the need for safeguards. Proposed safeguards included clear guidelines for use, technological modifications to protect privacy, meaningful consent processes, and, in Australian jurisdictions with human rights legislation, ensuring that VAPM is “assessed [as to] it’s human rights compliance” (Participant 11). Several agreed that additional “pilot” studies would be needed to test claims being made about the technology. One nurse raised the point that she would want to learn from “a mental health unit that’s using it, [in which] people have responded, [and in which] their experiences are positive” (Participant 4).

#### Is VAPM a Technological Solution to the Wrong Problem?

One consumer described VAPM as:

a solution to problems that shouldn’t have existed to begin with. That’s kind of, I think where I’m sitting, we’re providing solutions to problems that just shouldn’t be problems.

[Participant 7]

She elaborated by stating:

Oh, you won’t be getting a light shone in your eyes every 15 minutes. That’s that sounds amazing—but can’t we just stop doing it anyway?

This encapsulated a critique of the plan to rely on technology to fix issues that stem from what they view as poor clinical practice or systemic failures. Another consumer, who was trained as a nurse, noted that nurses do not need to shine a torch in someone’s eyes; she was taught to “shine the torch on the roof of the ceiling and you get an ambient light that that gives you enough to actually read obs, but not disturb the patient” (Participant 8). This concern relates to the earlier point, raised by consumer participants, that VAPM bypasses the need for genuine, therapeutic human interaction, which is the foundational element of mental health care.

#### Confusion Over the Specifics of VAPM

Most participants identified several aspects of VAPM that they felt were unclear, and several questions they would want answered before forming a view of the technology and its likely impact on the service system in which it would be used.

Because the workshop was held in a national context where VAPM had not been used, participants were not assessing a single, fully specified VAPM system. Limited technical detail about system configuration, performance (eg, false alarms), data flows, access controls, data retention, and workflow integration influenced how participants responded. Given the speculative nature of the discussion, participants had to reason from general principles, such as privacy and dignity, and draw analogies with other care contexts, such as in-person monitoring. At the same time, this uncertainty was itself salient to participants and contributed to the strong emphasis on transparency, independent evaluation, and governance safeguards as prerequisites for any trial or adoption.

Participant 6 worried that if the training datasets for the AI were biased (intentionally or unintentionally), the technology might be “more accurate for some people” than others (eg, men more than women), information which staff would not know because the workings are proprietary. Participant 3, the hospital manager whose service was considering this technology, stated that a key goal of their planned pilot was to determine if the technology was actually “going to be able to deliver what it can from an alerting perspective”—a matter which was unknown. Participant 9 (nurse academic) suggested that evidence would be needed to determine whether VAPM kept nurses “in the nurses station more and [on] the floor less,” and questioned whether monitoring would be restricted to nighttime use or applied more broadly. Participant 7 (consumer) questioned if it would be used only for a “couple of beds” or for the “whole ward.”

For most participants, therefore, views on the appropriateness of the technology in acute psychiatric care hinged on unresolved questions about the empirical basis for the technology, its scope and functionality, and questions about how it would be governed, such as what procedures were in place to protect patient privacy and prevent misuse.

### VAPM Has the Potential to Transform Not Just Augment Service Delivery

The potential service transformation created by the use of this sociotechnical system was another theme in the workshop, which the researchers subdivided into 3 themes: function creep, cost minimization and corporatization, and confusion about unresolved evidentiary and governance matters.

#### Risk of VAPM Function Creep

Some participants worried that the monitoring scope would quickly expand from passive overnight checks in some rooms to encompass day shifts, hallways, and potentially all consumer and patient rooms, eroding privacy and human interaction. Participant 8 (consumer) described a “slippery slope” in which allowing VAPM in some rooms today would mean “eventually they’ll just be in every room,” to the point “we stop having any interaction, any sort of relational space between clinical staff and the consumers.” A hospital manager (Participant 15) described the reality of function creep in health systems generally: “[t]he way systems work, the way organisations work, these things just happen without people thinking about it properly and putting the correct controls in place.”

#### VAPM as a Form of Cost Minimization and Privatization

A recurring fear by some participants was that VAPM would be used as a cost-cutting measure rather than a tool to improve care, with some participants concerned that it would justify a push for nurses to take on larger numbers of patients or consumers. Some participants worried that, in a risk-averse system with finance as a major driver, the technology might provide what a public health researcher described as “just... an excuse to reduce staff numbers to actually do things that will undermine the system improving, not actually support improvements” (Participant 5). A nursing academic (Participant 14) who supported its use in certain circumstances, similarly cautioned that it could be “used as an excuse to reduce the nursing resource and also that human interaction between nurses and consumers.” A broader concern was raised by Participant 11 about the role of the private technology sector in the development of health care technologies used in public health settings.

## Discussion

### Principal Findings

This study used a workshop with a range of key stakeholders to explore the potential implications of VAPM in acute psychiatric settings. The findings provide key insights into the impact VAPM might have, primarily for patients or consumers and nurses.

VAPM raised issues that sit within broader debates about surveillance technologies in health care, which are often justified in terms of safety and efficiency but raise concerns about consent, privacy, data governance, and the impact of monitoring on trust and therapeutic relationships [31]. Our findings reflect these tensions in a mental health inpatient context, highlighting how anticipated benefits are entangled with concerns about intrusion, misuse, and the conditions required for legitimate and acceptable use.

Participants who saw potential benefits emphasized patient safety and care efficiency, but these were framed tentatively and with caution, as potential benefits. Some tension emerged between groups: hospital managers and technologists broadly supported its testing and development; nurses and consumers varied somewhat but generally viewed VAPM negatively, with some qualifications. Most consumer participants were of the view that VAPM should be rejected, following its characterization as a form of intrusive surveillance that dehumanized patients and those who were supposed to care for them (for a similar viewpoint in the literature, see Powell et al [7]). Some participants, particularly nurses, saw potential use in specific circumstances and only if there was compelling empirical evidence to support its use, and ideally with patient consent. Most consumer and nursing participants articulated the view that the technology risked undermining nursing practice by replacing nursing functions and limiting the ability of nurses to develop therapeutic relationships. Across groups, the lack of robust pilot studies was a recurring concern, leaving many participants hesitant to endorse the technology, while its strongest critics rejected the premise of its use altogether.

These findings could be used to argue that these technologies should not be adopted due to the risks they pose to the privacy and dignity of patients. Alternatively, they could also be used to inform the design and implementation of these technologies to ensure that the sociotechnical system of VAPMs contributes not only to clinical and management outcomes but also to dignity, experience, and privacy outcomes for patients.

Regarding therapeutic interaction, participants were clear that VAPM should not replace human care. Nurses acknowledged the value of reducing intrusive overnight checks but emphasized that fewer face-to-face interactions might also limit opportunities for rapport and engagement. Although some were cautiously open to exploring alternatives that would minimize sleep disruption, nurses and consumers wanted clarity about how the technology would be used, how privacy would be safeguarded, and how such matters would be communicated to patients. Participants alluded to the cost minimization objective seeming to butt up against claims that VAPM would be an adjunct to—rather than replacement of—face-to-face care, echoing the observation of Griffiths et al [14] that “it is unclear how this adjunctive role is envisioned alongside the stated aim of cost reduction.” These concerns were often expressed by participants who explicitly or tacitly seemed to acknowledge that consumers and frontline staff are rarely the ultimate decision-makers in mental health hospitals.

In relation to privacy, VAPM was described as potentially less invasive if used in ways that reduce human intrusion and rely on abstracted physiological data rather than live video streams. From this view, VAPM could enhance care without reproducing the direct visual surveillance of closed-circuit television. However, participants cautioned that whether or not video feeds were actively viewed might matter little to patients, whose perceptions or experience of constant monitoring would still be experienced as intrusive.

Consent was a further complication, and our findings highlight challenges to individual autonomy, particularly within the coercive context of involuntary inpatient care. Some participants’ skepticism about opt-out arrangements and the fear that monitoring would continue regardless of refusal suggests that consent is, at best, a partial safeguard for VAPM in these settings. Consent processes may improve acceptability for some (particularly where monitoring is time-limited and genuinely optional), but they cannot be relied on as the primary ethical justification for deployment.

While some argued for an opt-in process, others noted that in mental health inpatient settings, where involuntary psychiatric intervention is common [32], it is unclear whether genuine consent for VAPM will be possible in some cases or legally required. Legal status does not map neatly onto decision-making capacity, which may fluctuate, but the conditions of admission, power imbalance, and role of risk-aversion in services could make refusal difficult in practice. Furthermore, opting out may not necessarily be respected or even feasible in a resource-constrained system. Gooding and Clifford [17] have even queried (tentatively) whether VAPM in mental health inpatient settings would conform with current law in some Australian jurisdictions, particularly if video is used. In the United Kingdom, the nongovernment organization Rethink Mental Illness argues that in addition to being required to meet medical-device regulatory requirements, VAPM should be conceptualized as “a form of restrictive practice that breaches people’s rights, with potential short and longer-term effects on people” [33]. These considerations also suggest that VAPM may (inadvertently) facilitate algorithmic paternalism [34], where algorithmic risk-management overrides a patient’s agency or autonomy under the guise of safety. An individual’s lack of agency over data captured from their most private moments, such as sleep or personal activities, may represent a fundamental breach of dignity that cannot be solved by a simple opt-in or opt-out checkbox. These concerns suggest that consent is, at best, a partial safeguard for VAPM in these settings. Consent processes may improve acceptability for some (particularly where monitoring is time-limited and genuinely optional), but they cannot be relied on as the primary ethical justification for deployment.

Uncertainty on matters of regulation and governance among the participants suggested that considerable planning and oversight would be needed before monitoring systems were trialed or adopted. Participants seemed to stress (and we agree) that any plans for implementation must go beyond minimum legal requirements to include consideration of social or ethical acceptance and perceived benefit among those most affected. On a related note, VAPM illustrates the insufficiency of perceived usefulness and perceived ease of use, the 2 pillars of the original technology acceptance model, which is used to explain whether, how, and why users adopt or reject a new technology [35]. Technology acceptance, particularly in health care, is not just about individual perceptions of usefulness and ease of use, but also about trust, perceived risk, and the social context in which the technology is introduced.

Again, such perspectives on implementation diverged along stakeholder lines. Consumers and nurses, whose daily experiences would be most directly affected, tended to be skeptical or resistant. They saw risks to therapeutic relationships and to their sense of agency and privacy. Technology vendors, by contrast, sought to promote their products by identifying problems to solve, and managers focused on organizational priorities rather than the details of nursing practice. Such contrasts highlight the importance of recognizing not only official intentions for new technologies but also the way they are interpreted and taken up.

On this latter point, there is some evidence from research on electronic health records that perceptions strongly shape adoption, where early concerns by stakeholders with electronic health records continued to influence professional practice long after implementation [36]. Management and technology providers may communicate their intention for a technology, but it is the users’ interpretation of the technology that plays a key role in how it is used and whether it is used as planned or not. Here, “users” are ostensibly nurses and other health practitioners at the ward level and could potentially extend to patients or consumers, though “users” seem an unsatisfactory description of this group. (On this latter point, Boetto [37] raises concerns in social care contexts that “end users” of AI are often envisaged as policymakers, managers, and practitioners, but not the service users who will ultimately experience its impacts and are best placed to identify risks and benefits.) Regardless, early perceptions such as those identified in this study can help us understand how end users and those affected will use and engage with the technology if implemented.

The theory of contextual integrity [38] may offer a useful lens for these concerns. Privacy, on this view, is defined by the appropriate flow of information within a specific context. What is appropriate is defined by context-specific information norms. Information norms that guide appropriate information flow can be mapped using the 5 parameters of actors (sender, receiver, and subject), information type, and transmission principle (which are constraints on information flow, such as consent). VAPM challenges established norms across several of these parameters by enabling more extensive data collection (eg, continuous rather than occasional checks), involving more actors (beyond the nurse at the door), and removing established principles of reciprocity and warning (patients can no longer see or hear when they are being observed). These shifts raise questions about whether such information flows are justified by reference to contextual values and whether less intrusive alternatives might achieve the same ends. Nissenbaum [39] suggests that, even where new technologies breach existing information norms, consideration should be given to whether the new information flows introduced by the technology serve the interests of different stakeholders, and whether they align with the ethical and political values of the specific context. Such an evaluation may result in breaches of existing information flows being viewed as justified. In mental health care, this question of alignment is especially difficult because ethical and political values are contested, particularly in inpatient settings involving involuntary psychiatric intervention, where competing views persist about whether risk and safety management should take precedence over the choices and rights of service users.

Applying contextual integrity also points toward possible design modifications. For instance, proactive notifications could alert patients when monitoring is active, or monitoring could be restricted to times when patients are asleep, thereby reducing unnecessary intrusion. Patients might also be given access to the data collected, for example, via a display screen. Such features potentially mitigate some of the privacy concerns raised in this study and afford patients a degree of control.

The erosion of digital trust [40] identified by participants is linked to the violation of contextual integrity. Trust in a mental health setting is built on the expectation that information remains within the boundaries of the clinician-patient relationship. VAPM challenges these established norms by involving new, often invisible actors, such as third-party technology vendors and data analysts. This in turn raises concerns about function creep and the commodification of patient data, that data collected for safety monitoring might eventually be used for purposes such as staff performance management or sold to create behavioral profiles. Establishing digital trust in VAPM, therefore, requires more than technical accuracy; it demands algorithmic accountability and transparent data stewardship that ensures information flows remain aligned with the ethical values of the psychiatric context.

Finally, there remains the question of how VAPM affects mental health itself. Paranoia and persecutory beliefs are common among people admitted to mental health inpatient settings [41], with evidence suggesting more than 70% of patients at first-episode psychosis experiencing some form of persecutory delusion [42]. Early evidence suggests that surveillance technologies may heighten such fears for some patients who have reported concerns that devices could “emit damaging rays” or “control them” [9], notwithstanding the potential benefits noted earlier. Future research is needed to assess whether purported benefits outweigh the potential negative psychological impacts of VAPM in these contexts.

### Implications for Policy and Practice

The findings of this study raise serious issues for future research, policy, and practice, which we summarize in Textbox 1.

Textbox 1. **Recommended steps for hospital managers, policymakers, and so on.**

- Start with an appraisal decision, not implementation: use structured deliberative processes (like this workshop) to decide whether video-algorithmic patient monitoring is appropriate in particular inpatient mental health settings.
- Define the “nonnegotiables” up front: identify minimum requirements relating to dignity, privacy, trust, and therapeutic relationships that must be met before video-algorithmic patient monitoring can be considered.
- Specify the proposed system and use-case before any decision: clarify what is being deployed (video vs abstracted data, alerting functions, where or when used, and who can access) so stakeholders are not evaluating an undefined intervention.
- Require independent evidence as a precondition: treat claims about safety, efficiency, and cost as provisional unless supported by independent, publicly available evaluation.
- Assess consent feasibility in involuntary contexts: determine whether meaningful consent or opt-out is possible in practice; if not, treat this as a major barrier requiring a higher threshold of justification.
- If proceeding, set governance first: establish binding rules on purpose limitation, oversight and accountability, transparency, data handling or retention, auditability, and safeguards against misuse and function creep, with meaningful consumer participation.

We elaborate on 3 points here.

First, given the contested and still limited evidence base, the findings support the need for independent, coproduced research that examines both the effects of VAPM and the conditions under which it might be regarded as legitimate and acceptable. Participants consistently questioned whether claimed benefits would be realized in practice, or whether they would be offset by risks to privacy, dignity, and therapeutic relationships. Independent, high-quality research that centers lived experience perspectives alongside frontline staff perspectives is therefore critical for informing any decision to proceed. Where VAPM is already in use, such evaluations should examine uptake across contexts, impacts on professional practice and ward culture, and the experiences of patients or consumers and staff. If a trial is contemplated, it should be treated as a research activity with transparent protocols and publicly reported outcomes, rather than a step toward routine adoption.

Second, participants made clear that decisions cannot be made about an ill-defined intervention. To reduce confusion and enable informed appraisal, any proposal to use VAPM should be accompanied by a clear description of the system and use-case: whether monitoring relies on video or abstracted data; when and where it is active (eg, selected beds vs whole wards and night-time vs broader use); measurable outcomes (eg, reducing false alerts, patient safety metrics, trust levels, incident reduction, and staff time spent with patients); who can access what information; and how data are stored, retained, and governed.

Processes for avoiding concerns about conflicts of interest must be clear [43]. On this latter point, one of the post-2021 studies reporting reduced self-harm following implementation was subsequently retracted [43]. While the retraction relates to an omitted conflict-of-interest declaration rather than an adjudicated finding of data error, it nonetheless highlights fragility in the VAPM evidence base and reinforces the importance of transparent disclosure and independent evaluation. As such, a transparent evaluation plan should be put in place to determine the accuracy of the technology and its impact on staff behavior, with results made public before any wider roll-out. By providing this clarity to all key groups, services can directly address the questions raised by staff, managers, and consumers in this study.

Finally, if VAPM is to be pursued, we would suggest that governance is established before implementation and should be guided by clear and collaboratively developed standards. Participants’ concerns point to the need for standards on purpose limitation, oversight and accountability, consent and choice (including how these operate in involuntary settings), auditability, and safeguards against function creep and secondary uses (including performance management). Policymakers and hospital administrators may wish to draw on participatory governance and co-design models used in digital mental health, including standing lived-experience advisory groups, coproduced implementation planning, and deliberative panels used to set acceptable data practices and safeguards [44-46]. Examples include Australia’s National Digital Mental Health Standards, which includes a “Consumer Partnership Principle” requiring service-user partnership across the planning, design, delivery, measurement, review, and evaluation of services [44], which can be adopted through practices like sustained lived-experience advisory groups that shape design and implementation over time [47].

### Strengths and Limitations

The strength of this study included the relatively diverse sample. This mix of participants—consumers, mental health nurses, health managers, and technology vendors—increased the diversity of views and minimized the risk of a single perspective dominating. Another strength was the coproduction process whereby the workshop was coplanned, co-designed, and coproduced with traditional academics and a consumer academic. This study appears to be the only empirical study on VAPM in mental health settings with consumer academic leadership and which provided for consumers to be equally important contributors to understanding the social, ethical, and legal implications of the technology. Finally, it could be considered a strength that this is an independent academic study with no vested interests, unlike most studies in the field (for an overview, see Griffiths et al [14]).

One limitation, regarding diverse consumer perspectives, is that we recruited only 3 consumers. Recruitment was constrained primarily by scheduling. Although additional consumers were invited to participate, they were not available at the workshop time. The 3 consumer participants had extensive experience in consumer advocacy roles, which may be associated with higher confidence in formal deliberation, greater familiarity with service systems and rights-based discourse, and an ability to articulate concerns in more conceptual terms than may be the case for others. Accordingly, the findings should not be treated as representative of the broader service user population, and the consumer accounts should be read as informed perspectives rather than a cross-section of typical inpatient views. However, our aim was exploratory: to elicit and compare stakeholder reasoning about the acceptability and implications of VAPM. On that basis, the consumer contributions were substantively valuable, foregrounding how privacy, coercion, dignity, and trust may be experienced and reasoned about in relation to VAPM. We do not claim statistical generalizability; instead, the findings offer analytic insights and issues for governance and practice that should be tested in future research with larger and more diverse consumer samples.

Purposive sampling was used, which supported a somewhat diverse sample but left the potential to amplify the authors’ biases, given the sample was effectively determined by author networks. Author positions may have influenced the framing of questions and interpretation. To mitigate this, we used reflexive practices across the study (including explicit efforts to set aside normative claims and a commitment to grounding themes in participants’ accounts and illustrative quotations). Nonetheless, positionality cannot be eliminated in qualitative research; hence, we have sought to make it transparent so readers can assess how the team’s perspectives may have shaped the analysis. The potential to amplify bias is somewhat offset by the consistency of the study aims (an exploratory study of viewpoints among people most likely to be impacted by the introduction of a technology), criteria (participants belonging to the main groups impacted), and epistemological basis of the research (valuing the qualitative input of those most affected in generating knowledge).

Response bias is a limitation. In this mixed-stakeholder deliberative workshop, participants may have tempered comments due to social desirability, professional norms around safety, or the presence of managers (in the case of nurses) or vendors. We mitigated this through independent moderation, neutral semistructured facilitation, and options for anonymous reporting, but some more critical or personal views may nonetheless be understated.

A further limitation is the time lag between data collection (December 2021) and publication. In the intervening period, the VAPM landscape—particularly in the United Kingdom—has evolved, with additional service evaluations and reviews published and wider use reported in some NHS mental health settings. There has also been increased public scrutiny and organized opposition, alongside policy attention, including an NHS-commissioned evidence review and work toward national guidance on vision-based monitoring. These developments mean that some issues raised in our workshop (eg, potential efficiency gains overnight) can now be compared against a larger, albeit still limited, evidence base, while other concerns (privacy, consent, dignity, and trust) remain live and contested in implementation debates.

As such, the study was somewhat hampered by the lack of empirical research at the time of the workshops, which had evaluated VAPM on its own terms, meaning that claims about its appropriateness could not be contrasted with claims about efficacy. Nor could we invite people who had encountered VAPM in the mental health care context, whether as patients, nurses, or hospital managers, given that it has not been trialed in Australian mental health inpatient settings to our knowledge, making the discussion largely hypothetical without the benefit of a specific VAPM definition or configuration for these inpatient settings. On the other hand, what this study suggests is that before technical development or empirical testing even takes place, the perspectives—if not leadership—of consumers and those working in mental health settings on the proposal should be sought.

Nevertheless, the workshop provided a valuable forum for persons likely to be impacted by VAPM to voice their views. There is value in deliberative input in the early stages of technology adoption or experimentation, particularly sociotechnical systems like VAPM. Our approach potentially offers a template for health services and researchers seeking to test views on proposed technologies, including for the purposes of risk mitigation, social acceptance, and safety.

### Conclusions and Future Research

This study highlights the considerable uncertainty surrounding the anticipated use of VAPM in mental health inpatient settings. Currently, anticipated risks are primarily articulated as subjective, as potential interference with privacy, dignity, and trust, while the claimed benefits lack robust and independent evaluation. Ethical and practical concerns voiced by consumers and frontline staff suggest that wide adoption would be premature, and some accounts that tend toward an ethics of care approach rejected the entire premise of VAPM. Any future trials must clearly define the technology’s purpose and mechanisms and be independently evaluated against not only safety and efficiency claims but also impacts on dignity, privacy, and therapeutic relationships. If VAPM is trialed, the findings of this study could inform both the technology configuration and model of care in which it is implemented. Crucially, research must be coproduced with those most directly affected—both staff and consumers, but particularly the latter, we would argue—to ensure that the technology serves the people it is intended to benefit.

## Supplementary material

10.2196/85518 Multimedia Appendix 1 Illustrative quotations from the deliberative workshop, organized by theme and subtheme.

## Abbreviations

AI: artificial intelligence
NHS: National Health Service
VAPM: video-algorithmic patient monitoring
