# Supplementary material for: Video-Algorithmic Patient Monitoring in Mental Health Inpatient Settings: Qualitative Study of Patient or Consumer, Clinician, and Vendor Perspectives
Source: JMIR Hum Factors. 2026 Jul 20;13:e85518. doi: 10.2196/85518 (PMC13384473; doi:10.2196/85518)
Supplement: Multimedia Appendix 1 [file humanfactors-v13-e85518-s001.docx]

Multimedia Appendix 1

**Table S1**.

| **Theme** | **Illustrative Quotation** |
| --- | --- |
| **Theme 1**  3.1 Contestation over the rationale for VAPM in mental health settings | ‘There's no electronic monitoring that will tell you that my hope was just crushed. Maybe you could have some electronic monitoring right down the chain when I was just about to die and maybe my, you know, my physiological measurements would start to change. It's too late. We [want] to be getting in early ... The only way you can know that we're thinking and feeling that is to talk with us and listen to us. And that's the beginning of the change, not the end of the line.’ (P2, consumer)  ‘… we've never had that facility in our [intensive care units] settings. We give them medication, we give patients, we sedate them, we send them to sleep. And how much do we really know about the danger?... I think this is an opportunity to augment our care … this is all about safety.’ (P15, hospital administrator) |
| **Theme 2** 3.2 VAPM will reshape care and relationships | ‘[T]here's also a question about “how does this change the way in which we relate to individuals, the way we relate to technology, the way in which we become dependent on technology?”’ (P11, consumer) |
| 3.2.1 VAPM may (de)humanise care depending on how it is used | ‘It’s potentially so dehumanizing for the person you're serving, but also the workforce that you need to be pinged to tell you to therapeutically engage with someone.’ (P4, nurse)  ‘If it's used well because nursing workload is really not about actually caring for the person, so we could probably use our time and resources to actually support the person in a more therapeutic way if the technology is doing the monitoring.’ (P14, nurse academic) |
| 3.2.2 Consent to monitoring | ‘Having the opportunity for consumers to nominate, you know, “are you comfortable with this with this technology being used whilst you're an inpatient?” And if not, you know, providing them with you with the alternatives … it would be 15 minute or hourly observations of a nurse coming in and checking on you and that physical interaction’. (P16, nurse academic) |
| **Theme 3**  3.3 Perceived harms of VAPM | ‘I feel pretty strongly about this. I think it's grotesque. I feel really scared and threatened by it, [in] many ways. I can imagine it will be misused and what it will be like as a consumer. I when I think about what it would be like for me and my experiences … I feel really angry as well because it feels to me the complete opposite direction that we should be heading for good care.’ (P2, consumer) |
| **Theme 4**  3.4 VAPM as observational augmentation for safety and risk reduction | ‘… to be able to sort of monitor that, that you know, that minimum physical health information would be really helpful. I think that the lack of overnight disruption would be great, too.’ (P13, nurse) |
| 3.4.1 Preventing rare but severe adverse events | ‘…one of the deep concerns I have always had is … [that] we give them medication, we give patients, we sedate them, we send them to sleep. And how much do we really know about the danger? Now, these are rare events, but as a clinician […] I think this is an opportunity to augment our care. […] So for me, this is all about safety.’ (P15, health service manager) |
| 3.4.2 Reducing disruption from routine observation | ‘From my experience, working with eating disorders, physical monitoring is a huge part. And I just think that this is possible, that we can do it because [so that we are] not just waking people up during the night… we were actually waking them up to do vital signs’. (P14, nurse academic) |
| **Theme 5**  3.5 VAPM has serious privacy implications | ‘…what this provides is around the clock 24 hour monitoring of them within a space that is otherwise private.’ (P13, nurse) |
| 3.5.1 Impacting privacy of individuals | ‘How do I know that it's turned off? And how do I know, for example, that some creepy person you shouldn't be working in mental health, but actually is, some creepy bloke on the nightshift hasn't turned it on to watch me because that's the kind of thing I'll be really worried about.’ (P2, consumer) |
| 3.5.2 Data protection | ‘… if it if it is all stored, where is it being stored and how long's it been stored for? […] There's be a huge amount of data being terabytes of tetrapods, wouldn't it?’ (P9, nurse academic) |
| **Theme 6**  3.6 VAPM requires appropriate governance | ‘… it just sharpens I think the need for making sure we've got good regulation and governance in place for this.’ (P11, consumer) |
| 3.6.1 Procedures and guidelines to support use of VAPM | ‘I think that if this was to be established, then there would really need to be some really strict guidelines around how we use it to supplement the care that we're providing, rather than using it as a replacement altogether.’ (P16, nurse) |
| 3.6.2 Safeguards in the adoption of VAPM | ‘… it depends on your protocol and what expectations you're putting on people and on the technology. So, you have to be thoughtful about how you introduce it and set it up.’ (P15, hospital manager) |
| 3.6.3 Is VAPM a technological solution to the wrong ‘problem’? | ‘But I feel like it's a solution to problems that shouldn't have existed to begin with. That's kind of, I think where I'm sitting, we're providing solutions to problems that just shouldn't be problems.’ (P7, consumer) |
| 3.6.4 Confusion over the specifics of VAPM | ‘And [I’m] confused as well in terms of the descriptions of the tech are always really vague, like I don't understand what makes it powered by artificial intelligence rather than just an algorithm like a risk assessment type thing.’ (P6, legal academic) |
| **Theme 7** 3.7 VAPM has potential to transform not just augment services | ‘I wonder how this, and just thinking again down the line with this stuff, so how does this intersect with like, you know, is it going to be a justification for less funding for nurses, patient ratios and whatnot?’ (P11, consumer) |
| 3.7.1 Risk of function creep | ‘… as soon as it does get started, “it's OK that that sounds reasonable. We'll use it at night.” But then there will be the benefit of, “you know, we can run a shorter, a smaller nursing profile if we don't have to watch people during a day”. And then these technologies end up being up in the corridors and then we end up with wristbands that actually identify us on whatever cameras anywhere in the room, in the in the in the hospital. It it's like a slippery slope to me.’ (P8, nurse) |
| 3.7.2 VAPM as cost minimisation and privatisation | ‘The first thing was, is this going to be used as an excuse to reduce the nursing resource?’ (P14, nurse academic) |
